# Findings From the International Lucid Dream Induction Study

**DOI:** 10.3389/fpsyg.2020.01746

**Published:** 2020-07-17

**Authors:** Denholm Jay Adventure-Heart

**Affiliations:** School of Psychology, The University of Adelaide, North Terrace Campus, Adelaide, SA, Australia

**Keywords:** lucid dreaming, lucid dream induction techniques, dream recall, reality test, sleep quality

## Abstract

The International Lucid Dream Induction Study (ILDIS) investigated and compared the effectiveness of five different combinations of lucid dream induction techniques including reality testing (RT), Wake Back to Bed (WBTB), the Mnemonic Induction of Lucid Dreams (MILD) technique, the Senses Initiated Lucid Dream (SSILD) technique, and a hybrid technique combining elements of both MILD and SSILD. Participants with an interest in lucid dreaming (*N* = 355) completed a pre-test questionnaire and then a baseline sleep and dream recall logbook for 1 week before practicing the lucid dream induction techniques for another week. Results indicated that the MILD technique and the SSILD technique were similarly effective for inducing lucid dreams. The hybrid technique showed no advantage over MILD or SSILD. Predictors of successful lucid dream induction included superior general dream recall and the ability to fall asleep within 10 min of completing the lucid dream induction techniques. Successful lucid dream induction had no adverse effect on sleep quality. Findings indicated that the techniques were effective regardless of baseline lucid dreaming frequency or prior experience with lucid dreaming techniques. Recommendations for further research on lucid dream induction techniques are provided.

## Introduction

In a lucid dream, the dreamer is aware that they are dreaming while the dream is still happening (LaBerge, 1985). According to a recent meta-analysis by Saunders et al. (2016), 55% of adults have experienced at least one lucid dream and 23% experience lucid dreams regularly (once per month or more). Recent research indicates that deliberate control is possible in approximately one third of lucid dreams (Soffer-Dudek, 2020). Examples include changing location and deliberately waking up (LaBerge and Rheingold, 1991; LaBerge and DeGracia, 2000; Love, 2013; Mota-Rolim et al., 2013). Lucid dreaming has many potential benefits and applications, such as treatment for nightmares (Spoormaker and Van Den Bout, 2006; Lancee et al., 2010; Holzinger et al., 2015), improvement of physical skills and abilities through dream rehearsal (Erlacher and Schredl, 2010; Stumbrys et al., 2016), creative problem solving (Stumbrys and Daniels, 2010), and research opportunities for exploring mind-body relationships and consciousness (see Hobson, 2009). However, to date the effects reported in most studies have been weak and inconsistent, and more research is needed into the applications of lucid dreaming (Baird et al., 2019; de Macêdo et al., 2019).

Many techniques exist for inducing lucid dreams (see Tholey, 1983; LaBerge and Rheingold, 1991; Stumbrys et al., 2012; Love, 2013). These techniques have been organized by Stumbrys et al. (2012) according to three broad categories. *Cognitive techniques* include mental exercises that increase the likelihood of lucid dreaming. The two most widely researched cognitive techniques are *reality testing* (RT; Tholey, 1983; LaBerge and Rheingold, 1991) and the *Mnemonic Induction of Lucid Dreams* (MILD) technique (LaBerge, 1980; LaBerge and Rheingold, 1991). RT involves examining one’s environment and then performing a reliable test that differentiates between waking and dreaming, repeatedly throughout the day. The rationale is that if RT becomes habitual, it will eventually be performed while dreaming, triggering lucidity. The MILD technique involves creating a prospective memory intention to remember that one is dreaming by repeating the phrase “next time I’m dreaming, I will remember I’m dreaming” (or some variation). The MILD technique is performed during a brief awakening after 5 or so hours of sleep. Indeed, waking up after several hours of sleep for the purpose of lucid dream induction is itself a technique, known as *Wake Back to Bed* (WBTB; LaBerge and Rheingold, 1991). When successful, the MILD technique triggers lucidity during subsequent REM sleep. *External stimulation techniques* involve stimuli such as flashing lights presented during REM sleep that can be incorporated into dreams, serving as cues that trigger lucidity. *Miscellaneous techniques* include lucid dream inducing drugs and supplements (see LaBerge, 2004; see also Yuschak, 2006).

Stumbrys et al. (2012) identified 35 empirical studies on lucid dream induction techniques in a systematic review. Most (24) were field studies, with the others conducted in sleep laboratories (11). Stumbrys et al. (2012) evaluated these studies using a methodological quality checklist developed by Downs and Black (1998) and found that most (60%) were of poor methodological quality. The others were classified as moderate quality. More than half of the studies were unpublished Ph.D. dissertations or otherwise not published in peer-reviewed journals. All studies showed poor external validity. Participants were mostly university students or self-selected and highly experienced lucid dreamers. Most lucid dreaming studies are also limited by small sample sizes, lack of random allocation, failure to investigate variables that operationalize the way in which techniques were practiced (e.g., number of technique repetitions), and inconsistent operationalization of lucid dreaming rates (see Aspy et al., 2017 for a more detailed discussion). These widespread limitations are a major impediment to lucid dream research and make it difficult to compare the effectiveness of techniques across studies.

Several additional lucid dream induction studies have been published since the publication of Stumbrys et al. (2012). Taitz (2011) found that daily RT for 2 weeks was ineffective. Poor success rates were reported in laboratory studies of external stimulation (flashing lights and vibration; Franc et al., 2014) and transcranial direct current stimulation (tDCS) to the dorsolateral prefrontal cortex (DLPFC) during REM sleep (Stumbrys et al., 2013). Dyck et al. (2017) found that keeping a dream diary, RT, and a combined WBTB and affirmation technique were ineffective. In a study by Konkoly and Burke (2019), 19 participants performed RT, MILD, and the Wake-Induced Lucid Dream technique (WILD). However, the authors did not provide statistics to indicate how effective this training program was except that 39 lucid dreams were reported. Saunders et al. (2017) found that a greater proportion of participants who practiced several techniques over a 12-week period (including RT, MILD and WBTB) experienced lucid dreaming compared to a control group (45 vs. 6%). However, the frequency of lucid dreaming is unclear. Kumar et al. (2018) reported a low success rate (at most 6% of days had lucid dreams) for Tholey’s combined technique, which involves regular reality tests combined with autosuggestion and intention to have a lucid dream (Tholey, 1983). Sparrow et al. (2018) found that the drug Galantamine was effective for inducing lucid dreams. However, results do not permit calculation of lucid dreaming rates. LaBerge et al. (2018) found that lucid dreaming occurred on 42% of nights when participants ingested 8 mg of Galantamine in addition to practicing the MILD technique, and in most cases, using an external stimulation device (flashing light). A success rate of 14% was reported for a control condition involving the same techniques but with placebo pills.

The National Australian Lucid Dream Induction Study (NALDIS; Aspy et al., 2017) provided a thorough investigation into RT, MILD and WBTB using a highly diverse sample of Australian participants (*N* = 169). During Week 1, participants recorded baseline dream recall rates and were then randomly allocated to one of three experimental groups for Week 2. Because RT, WBTB and MILD are often used in combination, and in the interests of identifying a maximally effective approach to lucid dream induction, an additive approach in which groups involving RT only (*RT only* group), RT and WBTB (*RT* + *WBTB* group) and RT, WBTB, and MILD (*RT* + *WBTB* + *MILD* group) were compared. A significant increase in lucid dreaming was observed in the *RT* + *WBTB* + *MILD* group, with lucid dreaming reported on 17.4% of nights in Week 2 compared to 9.4% of nights in Week 1. No significant changes in lucid dreaming frequency were observed in the other two groups. However, although RT was ineffective when practiced in isolation, it remained uncertain whether RT contributed to the significant increase in lucid dreaming rates observed in the *RT* + *WBTB* + *MILD* group. This is important because RT is a burdensome practice, and if ineffective, it would be better to simply practice WBTB and MILD. Higher general dream recall was a significant predictor of lucid dreaming following practice of the MILD technique. However, the strongest predictor of lucid dreaming was the amount of time taken to fall back asleep after completing the MILD technique. Lucid dreaming was experienced on 45.8% of occasions when participants completed the MILD technique and then fell asleep within 5 min. A likely explanation is that returning to sleep quickly makes it more likely that the MILD intention will persist into REM sleep and trigger lucidity.

The biggest impediment to research into the potential benefits and applications of lucid dreaming is the lack of effective and reliable lucid dream induction techniques. Despite a reduction of research interest in lucid dream induction over the past few decades (Stumbrys et al., 2012), many promising avenues for research remain. Numerous lucid dream induction techniques have been developed by lucid dreaming enthusiasts but have not been investigated scientifically. One promising example is the cognitive technique known as the *Senses Initiated Lucid Dream* (SSILD) technique (the double “S” in the acronym is intentional; Gary Zhang, 2013). The SSILD technique involves waking up after approximately 5 h of sleep (as with MILD) and then repeatedly shifting one’s attention between visual, auditory, and physical sensations before returning to sleep. The International Lucid Dream Induction Study (ILDIS) aimed to investigate the effectiveness of the SSILD technique and address unanswered questions from the NALDIS about the effectiveness of the MILD technique when practiced alone compared to when practiced in combination with RT. The ILDIS also aimed to compare two different types of RT and examine the effectiveness of a hybrid technique combining elements of both MILD and SSILD. Recruitment took place during a media release and subsequent media coverage that occurred when the NALDIS was published. The following hypotheses were tested:

• It was hypothesized that general dream recall rates would be positively correlated with lucid dreaming frequency at both pre-test and during Week 2.
• It was hypothesized that Week 2 lucid dreaming rates would be significantly higher than Week 1 lucid dreaming rates.
• It was hypothesized that lucid dreaming rates would be significantly higher when participants took 5 min or less to fall asleep after practicing lucid dreaming techniques compared to when they took more than 5 min to fall asleep.

## Materials and Methods

### Participants

An initial sample of 1618 participants completed the pre-test questionnaire. A total of 843 participants continued to complete Week 1 of the study and 355 participants completed Week 2. In the final sample there were 190 (53.5%) females, 162 (45.6%) males and 3 (0.9%) “other.” Mean age was 35.3 (*SD* = 12.4, range: 18–84). Most participants (*n* = 255) were employed non-students (71.8%), with 69 (19.4%) students and 31 (8.7%) unemployed or retired. Just over half of participants (54.9%) reported prior experience with lucid dream induction techniques. Only six participants (1.7%) had participated in prior lucid dreaming research. Participants reported *M* = 1.1 lucid dreams in the month prior to commencing the study (*SD* = 2.4, range: 0–28). Participants heard about the study from a wide range of sources that directed them to the present author’s website, where they could sign up to participate. Sources included: 183 (51.6%) from Facebook; 83 (23.4%) from other internet sources (e.g., email lists and social media); 40 (11.3%) from newspaper articles; 28 (7.9%) from a friend; 18 (5.1%) from radio interviews; and 3 (0.9%) from a television interview with the author. Country of residence was: 111 in United States (31.3%); 76 in Australia (21.4%); 26 in United Kingdom (7.3%); 25 in Canada (7.0%); 14 in Germany (3.9%); 9 in Mexico (2.5%); and 94 in a wide variety of other countries (26.5%). Participants were excluded from the study if they had been diagnosed with any kind of mental health disorder, sleep disorder, or neurological disorder; suspected they *might* have one of these disorders; were experiencing a traumatic or highly stressful life event that was interfering with their sleep; suffered from persistent insomnia or were unable to keep a regular sleep schedule; had experienced sleep paralysis more than once in the past 6 months; found it unpleasant to think about their dreams; or were under 18 years of age. No material incentive was offered. This study was granted ethics approval by the School of Psychology Human Research Ethics Subcommittee at the University of Adelaide. Participants were given an information sheet and then gave informed consent prior to participating.

### Materials

Materials included a pre-test questionnaire, logbooks for Week 1 and Week 2, and technique instructions documents. All pre-test, Week 1 logbook and Week 2 logbook measures were hosted online using the survey management website *Survey Monkey*. Instructions were sent via email. In the present paper, pre-test variables are identified by a capital “P” and logbook variables by a capital “L.”

#### Pre-test Questionnaire

Participants indicated their gender, age, occupation, how they heard about the study, their country of residence, and if they had ever participated in a scientific study on lucid dreaming techniques. Retrospective general dream recall was operationalized as *Dream Recall Frequency* (DRF; the percentage of days on which there was dream recall) and measured by asking “How many days during the last week did you remember your dreams from the previous night?” (*P DRF*). Response options ranged from “0 days” to “7 days.” Retrospective lucid dreaming rates were operationalized as *Dream Count* (*L DC Lucid per month*; the number of dreams recalled over the past month) and assessed using a question adapted from Brown and Donderi (1986) *Sleep and Dream Questionnaire* (SDQ): “Lucid dreams are those in which a person becomes aware of the fact that he or she is dreaming while the dream is still ongoing. For example: ‘I was in England talking to my grandfather when I remembered that (in real life) he had died several years ago and that I had never been to England. I concluded that I was dreaming and decided to fly to get a bird’s eye view of the countryside…’ Please estimate the number of lucid dreams you have had in the past month.” Response options ranged from 0 to 30 or “more than 30” (scale unit = 1, range: 0–20). Participants were asked “Have you ever tried to have lucid dreams by learning and then practicing a lucid dreaming technique?” (*P Lucid tech prior*; “yes” or “no”). Participants were asked, “How often have you practiced a lucid dreaming technique recently (in the past several months)?” (*P Lucid tech freq*). Response options from Schredl (2004) widely used dream recall measure were used (0 = never; 1 = less than once a month; 2 = about once a month; 3 = two or three times a month; 4 = about once a week; 5 = several times a week; and 6 = almost every morning). Responses were converted to the approximate number of days per week using the following class means: 0 = 0; 1 = 0.125; 2 = 0.25; 3 = 0.625; 4 = 1.0; 5 = 3.5; 6 = 6.5.

#### Logbooks

Participants wrote the date for each logbook entry. This information was used to calculate the number of days taken to complete all seven logbook entries (*L Days to complete log*). The total number of logbook entries was also counted (*L Total log entries*). Participants reported whether they could recall anything specific about their dreams from the preceding night and provided brief titles for each dream they could recall. Using this information, general dream recall was operationalized as both *Dream Recall Frequency* (*L DRF*; the percentage of days on which there was dream recall) and Dream Count (*L DC per day*; the number of dreams recalled each day). Participants also rated how much content they could recall from each dream according to four categories, operationalizing dream recall as *Dream Quantity* (*L DQ*). The measure was developed by Aspy (2016) and is based on an earlier measure developed by Reed (1973). Category ratings are converted to numerical values (“Fragmentary” = 1, “Partial” = 2, “Majority” = 4, “Whole” = 8) and summed (higher scores indicate superior dream recall). The number values 1, 2, 4, and 8 reflect the proportionate increase in dream content associated with the category labels and descriptions, based on qualitative data collected by Reed (1973). Lucid dreaming was operationalized as DRF (*L DRF Lucid*; the percentage of mornings on which lucid dreaming was reported) using the following question: “Did you have any lucid dreams last night? (Lucid dreams are those in which a person becomes aware of the fact that he or she is dreaming while the dream is still ongoing)” (“yes” or “no”). DRF was used instead of DC because participants were unsure of how many lucid dreams they had in some cases, and in other cases lost and regained lucidity within the same dream.

Participants estimated their total time asleep (*L Time asleep*): “How much time in total do you think you spent sleeping last night? hours, minutes.” Participants rated their subjective sleep quality (*L Sleep quality*): “On a scale of 1–5, what was the overall quality of your sleep last night?” (1 = “terrible,” 2 = “poor,” 3 = “okay,” 4 = “good,” 5 = “excellent”). Participants indicated how tired they felt on waking when they were finished sleeping (*L Tiredness on waking*): “On a scale of 1–5, how tired do you feel this morning?” (1 = “not at all tired,” 2 = “slightly tired,” 3 = “somewhat tired,” 4 = “quite tired,” 5 = “very tired”). Participants indicated their level of sleep deprivation from the previous day (*L Sleep dep yesterday*): “On a scale of 1–5, how sleep deprived were you yesterday?” (1 = “not at all,” 2 = “slightly,” 3 = “somewhat,” 4 = “quite,” 5 = “very”). This measure was included to assess any potential effect of sleep deprivation on lucid dream induction, e.g., due to a REM rebound effect.

The Week 2 logbooks included additional measures related to lucid dreaming technique practice. All participants were asked “Did you turn on the light when the alarm woke you up to do the lucid dreaming technique?” (*L Light on when awoke*; “yes” or “no”); “Did you get out of bed (including if you went to the toilet) when the alarm woke you up to do the lucid dreaming technique?” (*L Out of bed when awoke*; “yes” or “no”); “How long (approximately) did you spend on doing the technique? minutes.” (*L Technique min*); “Did you fall asleep while you were still trying to do the technique?” (“yes” or “no”) (*L Asleep during technique*); and “If you answered “no” to the above question, how long (approximately) did it take for you to get to sleep after you stopped doing the technique? minutes.” (*L Min back to sleep*). Participants who practiced RT (Groups 2 and 3) were asked “How many reality tests did you perform yesterday?” (blank space provided) (*L Reality tests*). Participants in Groups 1, 2, 3, and 4 that all involved the MILD technique were asked “How many times (approx.) did you repeat “next time I’m dreaming, I will remember I’m dreaming” after the alarm woke you up?” (*L MILD phrase repetitions*). Participants in Group 5 who practiced the SSILD technique were asked “How many fast and slow cycles did you do? Fast, Slow.” (*L Fast cycles* and *L Slow cycles*). Participants in Group 6, which involved the hybrid MILD and SSILD technique, were asked “How many cycles did you do after the alarm woke you up?” (*L Hybrid technique cycles*).

#### Lucid Dream Induction Technique Documents

All participants were advised to print their lucid dream induction technique instructions, keep them beside the bed, spend a full hour familiarizing themselves with them before commencing the study, practice their techniques at least once during the day to ensure understanding, and to revise the instructions directly before bed each night. All participants were instructed to set an alarm 5 h after going to bed, to place the alarm somewhere that would require getting out of bed to turn it off, and to then practice their assigned “Nighttime Technique” when the alarm went off. Based on findings from the NALDIS, the importance of falling asleep quickly after practicing the techniques was emphasized. Participants were advised that if they were falling asleep too quickly, they could try turning the lights on for a few minutes and reading over the technique instructions to increase wakefulness. They were advised to keep the lights off, put the alarm next to their bed, and use a quieter alarm tone if they had trouble returning to sleep. All participants were given instructions on how to perform an RT if they suspected they were dreaming but were not sure. Participants were told not to practice RT during the day except for participants in Group 2 and Group 3 (see section “Group 2: MILD + WBTB + RT Breath” and section “Group 3: MILD + WBTB + RT Hands”). Participants were also given information and advice about sleep paralysis (see LaBerge and Rheingold, 1991; Sleep Paralysis Information Service, 2013; University of Waterloo, 2013). Instructions that were specific to each group are provided below.

##### Group 1: MILD + WBTB (No RT)

Participants in this group were given a “Nighttime Lucid Dreaming Technique” document that contained instructions for the MILD technique. This involved recalling a dream from directly prior to waking up (or alternatively, any other recent dream), laying down comfortably, and then repeating the phrase “next time I’m dreaming, I will remember I’m dreaming.” The importance of strong intention was emphasized. Participants were told to simultaneously visualize being back in the dream they had recalled and noticing something unusual that causes them to realize they are dreaming. They were advised to continue until they felt their intention was set.

##### Group 2: MILD + WBTB + RT Breath

These participants were given the same MILD instructions as Group 1. They were also provided with instructions for performing a minimum of 10 inhalation RT per day. This involves closing one’s lips and then attempting to inhale through the mouth, which is possible in dreams but not while awake (see Aspy et al., 2017).

##### Group 3: MILD + WBTB + RT Hands

This group was given a different kind of RT from Group 2, which involves attempting to push the fingers of one hand through the palm of the other. This was chosen because it is one of the most widely practiced RT. The ability to push the fingers through the palm indicates that one is dreaming. Participants were advised to also inspect their hands for anomalies during each test.

##### Group 4: MILD + WBTB (No RT)

Instructions for this group were the same as the instructions for Group 1, with no modifications. The decision to include a second *MILD* + *WBTB (no RT)* group in Cohort 2 was based on the fact that some participant sample characteristics changed over time during the recruitment process (see section “Preliminary Analyses”). The inclusion of a second *MILD* + *WBTB (no RT)* group in Cohort 2 permitted valid comparison of the MILD and SSILD techniques.

##### Group 5: SSILD + WBTB (No RT)

Instructions for the SSILD technique were designed with consultation from the creator of the technique. It was explained that the technique works by conditioning the mind and body into a subtle state that is optimized for lucid dreams to occur, and that it involves performing several “cycles” that each involve the following three steps:

*Step 1. Focus on Vision*: Close your eyes and focus all your attention on the darkness behind your closed eyelids. Keep your eyes completely still and totally relaxed. You might see colored dots, complex patterns, images, or maybe nothing at all. It doesn’t matter what you can or cannot see – just pay attention in a passive and relaxed manner and don’t “try” to see anything.
*Step 2. Focus on Hearing*: Shift all of your attention to your ears. You might be able to hear the faint sounds of traffic or the wind from outside. You might also be able to hear sounds from within you, such as your own heartbeat or a faint ringing in your ears. It doesn’t matter what, if anything, you can hear – just focus all of your attention on your hearing.
*Step 3. Focus on Bodily Sensations*: Shift all of your attention to sensations from your body. Feel the weight of the blanket, your heartbeat, the temperature of the air, etc. You might also notice some unusual sensations such as tingling, heaviness, lightness, spinning sensations, and so on. If this happens simply relax, observe them passively and try not to get excited.

Participants were instructed to first perform four fast cycles (2 or 3 s on each step) and then four to six slow cycles (approximately 20 s on each step). They were told not to count the number of seconds, and that it is important to complete at least four slow cycles. Participants were instructed to fall asleep as normal after completing six slow cycles.

##### Group 6: SSILD/MILD Hybrid + WBTB

Participants were asked to do only four to six slow cycles (no fast cycles) and to repeat the MILD phrase “next time I’m dreaming, I will remember I’m dreaming” every time they switched to a new sensory modality. The importance of strong intention was emphasized. Participants were not asked to recall dreams or do any visualization.

### Procedure

The ILDIS was conducted entirely via the internet, allowing people from around the world to complete the study at home. Participants were directed to a web page about the ILDIS using a URL included in a range of media items (see section “Participants”), where they read the information sheet and completed the pre-test questionnaire. Participants were sent emails with instructions and web URLs for accessing the Week 1 logbooks hosted on *Survey Monkey*. Participants were instructed to complete each logbook entry immediately upon waking, and to not practice any lucid dreaming techniques during Week 1. Participants were given instructions on how to improve their dream recall during both Week 1 and Week 2. Upon completing Day 7 of the Week 1 logbook, participants were sent further instructions, lucid dream induction technique documents, and additional web URLs to access the Week 2 logbooks. Participants were asked to practice the techniques and make logbook entries on consecutive days if possible, but not to practice the techniques if they were sleep deprived. They were instructed to make up for any skipped days at the end. Once sufficient sample sizes had been achieved for the three groups in Cohort 1 (permitting comparison of MILD practiced with and without two kinds of RT), the author began randomly allocating new participants to the three groups in Cohort 2 (permitting comparison of MILD with SSILD and the SSILD/MILD hybrid technique, all without RT). NALDIS group sizes were used as a guide in determining adequate group sizes in the ILDIS.

## Results

### Preliminary Analyses

Analyses were conducted using IBM SPSS 26 for Windows. Non-parametric tests were used in all cases because most variables were non-normally distributed. There was no significant difference in the proportions of participants who were employed non-students, students, and unemployed or retired who did and did not complete the full study: χ^2^(2, *N* = 1615) = 3.43, *p* = 0.180, *V* = 0.05. The proportion of participants who reported prior experience with lucid dreaming techniques at pre-test was significantly higher for participants who completed the full study (54.9%) compared to those who did not (43.5%): χ^2^(1, *N* = 1615) = 14.59, *p* = 0.001, *V* = 0.10. Mann-Whitney tests indicated that participants who completed the full study had significantly higher general dream recall rates and *P Lucid tech freq* at pre-test. These findings and descriptive statistics for pre-test variables are presented in Table 1.

**TABLE 1:** Descriptive statistics for pre-test variables with Mann-Whitney tests for differences between participants who did and did not complete the full study.

**Pre-test variable**	**Completed full study (*N* = 355)**	**Did not complete full study (*N* = 1260)**	**Mann–Whitney test**		
	***M* (*SD*)**	***M* (*SD*)**	***Z***	***p***	***r***
P Age	35.3 (12.4)	34.5 (12.1)	1.00	0.318	0.03
P DRF	42.8%(28.5%)	38.4%(28.0%)	2.34	0.019	0.06
P DC Lucid per month	1.1 (2.4)	1.5 (3.7)	0.53	0.593	0.01
P Lucid tech freq	0.4 (1.1)	0.3 (1.0)	2.17	0.030	0.05

*P, pre-test variable.*

There were no significant differences between Cohort 1 and Cohort 2 on any pre-test, Week 1 or Week 2 variables except for: *P Age* (Cohort 1 *M* = 32.4, *SD* = 10.2; Cohort 2 *M* = 37.2, *SD* = 13.4; *Z* = 3.28, *p* = 0.001, *r* = 0.17); Week 1 *L Sleep quality* (Cohort 1 *M* = 3.6, *SD* = 0.5; Cohort 2 *M* = 3.4, *SD* = 0.5; *Z* = 2.10, *p* = 0.036, *r* = 0.11); and Week 1 *Days to complete log* (Cohort 1 *M* = 7.8, *SD* = 1.5; Cohort 2 *M* = 7.9, *SD* = 6.8; *Z* = 3.95, *p* = 0.001, *r* = 0.21). There were no significant differences between the three groups within Cohort 1 or within Cohort 2 on these variables. Non-significant test results are not reported for the sake of brevity. Descriptive statistics and Wilcoxon tests of differences between Week 1 and Week 2 logbook variables are presented in Table 2. Results showed that participants reported significantly higher *L Time asleep* and significantly lower general dream recall rates, *L Tiredness on waking* and *L Total log entries* in Week 2 of the study compared to in Week 1.

**TABLE 2:** Descriptive statistics and Wilcoxon tests for differences between week 1 and week 2 logbook variables for participants who completed the full study.

**Logbook variable**	**Week 1 (*N* = 355)**	**Week 2 (*N* = 355)**	**Wilcoxon test**		
	***M* (*SD*)**	***M* (*SD*)**	***Z***	***p***	***R***
L DRF	85.0%(17.9%)	79.8%(28.1%)	2.73	0.006	0.15
L DC per day	1.9 (1.0)	1.7 (1.1)	4.21	<0.001	0.22
L DQ	5.7 (4.4)	5.6 (5.1)	0.50	0.621	0.03
L Time asleep	7.4 (0.9)	7.7 (1.0)	5.14	<0.001	0.27
L Sleep quality	3.5 (0.5)	3.4 (0.6)	1.44	0.150	0.08
L Tiredness on waking	2.34 (0.6)	2.27 (0.8)	2.09	0.036	0.11
L Sleep dep yesterday	1.9 (0.7)	2.0 (0.8)	0.75	0.456	0.04
L Total log entries	6.9 (0.4)	4.6 (2.2)	13.19	<0.001	0.70
L Days to complete log	7.9 (5.4)	7.7 (5.8)	0.85	0.396	0.05

*L, logbook variable.*

### Relationships With Lucid Dreaming

It was hypothesized that general dream recall rates would be positively correlated with lucid dreaming frequency at both pre-test and during Week 2. Spearman rho non-parametric correlations supported the hypothesis and are presented in Table 3. All pre-test general dream recall variables were related to *P DC Lucid per month*. Correlations between pre-test general dream recall variables and Week 2 *L DRF Lucid* were weaker but still significant in all cases. All Week 2 general dream recall variables were significantly correlated with both *P DC Lucid per month* and Week 2 *L DRF Lucid*, with the relationships being stronger with Week 2 *L DRF Lucid* in all cases. This pattern of findings highlights the imperative to not treat retrospective and logbook variables of dream recall as equivalent (see Aspy et al., 2017; see also Aspy, 2016). A weak correlation was observed between *P Lucid tech freq* and *P DC Lucid per month* but not with Week 2 *L DRF Lucid*. Pre-test and Week 2 lucid dreaming rates were positively correlated. *P Age* was weakly correlated with *P DC Lucid per month* but not with *L DRF Lucid.*

**TABLE 3:** Spearman rho non-parametric correlations between pre-test and week 2 lucid dreaming rates and other pre-test and week 2 variables.

	**P DC Lucid per month**	**L DRF Lucid (week 2)**
P DC Lucid per month	–	0.38**
P Lucid tech freq	0.18**	–0.06
P Age	0.05*	0.10
P DRF	0.33**	0.14*
L DRF (Week 2)	0.15**	0.22**
L DC per day (Week 2)	0.16**	0.31**
L DQ (Week 2)	0.21**	0.30**

P, pre-test variable; L, logbook variable. Correlations with pre-test variables and L DRF were calculated using mean Week 2 L DRF Lucid values for each participant. Correlations with all other logbook variables were calculated using individual daily observations and are point-biserial. *p = 0.05, **p = 0.01.

### Lucid Dream Induction

It was hypothesized that Week 2 lucid dreaming rates would be significantly higher than Week 1 lucid dreaming rates. This hypothesis was supported. Dependent samples Wilcoxon tests showed that Week 2 *L DRF Lucid* was significantly higher for all participants combined and for each of the six Week 2 groups, with medium to large effect sizes in all cases. These results are presented in Table 4. Logbook day was significantly related to *L DRF Lucid* in both Week 1 [χ^2^(6) = 13.21, *N* = 2448, *p* = 0.040, *V* = 0.07] and Week 2 [χ^2^(6) = 28.51, *N* = 1647, *p* = 0.001, *V* = 0.13], with the tendency for *L DRF Lucid* to decrease slightly over time. Because of the significant difference in *L Total Log entries* between Week 1 (*M* = 6.9) and Week 2 (*M* = 4.6) noted in section “Preliminary Analyses,” there were concerns that the Week 2 *L DRF Lucid* rate may be inflated compared to the Week 1 *L DRF Lucid* rate. To control for this issue, analyses were repeated comparing mean *L DRF Lucid* rates based on only the first four logbook days of Week 1 and Week 2. *L DRF Lucid* was again significantly higher for all participants combined and for participants in all six of the Week 2 groups, confirming the effectiveness of the techniques. Independent samples Kruskal-Wallis tests showed that there were no significant group differences within Cohort 1 (χ^2^ = 1.51, *p* = 0.471, *r* = 0.06) or Cohort 2 (χ^2^ = 4.16, *p* = 0.125, *r* = 0.11) in Week 2 *L DRF Lucid.* The combined *L DRF Lucid* rate for the two MILD + WBTB groups that did RT during the day (*n* = 88, *M* = 12.1%, *SD* = 20.4%) was compared to the combined rate for the two MILD + WBTB groups that did not do RT during the day (*n* = 118, *M* = 19.4%, *SD* = 27.8%). Results from a Mann-Whitney test were non-significant (*Z* = 1.94, *p* = 0.052, *r* = 0.14).

**TABLE 4:** Differences between week 1 and Week 2 lucid dreaming rates for all participants combined and for each of the six week 2 groups.

**Week 2 group**	**L DRF Lucid**			**Wilcoxon test**		
	**Week 1 *M* (*SD*) (%)**	**Week 2 *M* (*SD*) (%)**	**Improvement (%)**	***Z***	***p***	***r***
All participants combined (*N* = 355)	5.3 (13.4)	15.8 (25.2)	199.0	8.37	<0.001	0.44
Group 1: MILD + WBTB (no RT) (*n* = 54)	6.5 (16.4)	18.4 (28.7)	185.7	3.12	0.002	0.42
Group 2: MILD + WBTB + RT Breath (*n* = 44)	1.0 (3.6)	10.8 (14.0)	1006.1	3.74	<0.001	0.56
Group 3: MILD + WBTB + RT Hands (*n* = 44)	5.2 (14.5)	13.4 (25.3)	157.3	2.68	0.007	0.40
Group 4: MILD + WBTB (no RT) (*n* = 64)	6.8 (14.7)	20.2 (27.2)	198.0	3.99	<0.001	0.50
Group 5: SSILD + WBTB (no RT) (*n* = 76)	4.7 (10.8)	16.9 (27.2)	258.9	4.43	<0.001	0.51
Group 6: SSILD/MILD Hybrid + WBTB (*n* = 73)	6.3 (15.0)	13.3 (23.3)	109.6	2.71	0.007	0.32

*L, logbook variable.*

### Relationships With Technique Practice Variables

Relationships between *L DRF Lucid* and variables that operationalize the way in which the lucid dreaming techniques were practiced were assessed using Spearman rho non-parametric correlations and are presented with descriptive statistics in Table 5. All correlations were non-significant except for a weak correlation between *L Fast cycles* performed by participants in Group 5: SSILD + WBTB (no RT) and *L DRF Lucid*. The results remained non-significant in all cases when correlations were repeated for each group individually, except for a weak negative correlation observed between *L Technique min* and *L DRF Lucid* in Group 5: SSILD + WBTB (no RT) (*r*_*s*_ = -0.16, *p* = 0.013, *n* = 256).

**TABLE 5:** Spearman rho non-parametric correlations between Week 2 lucid dreaming rates and variables that operationalize the way in which the lucid dream induction techniques were practiced.

	***M* (*SD*)**	**Correlation (*r*_*s*_) with L DRF lucid**
L Reality tests (Groups 2 and 3)	4.1 (4.4)	–0.04
L MILD phrase repetitions (Groups 1, 2, 3, and 4)	13.6 (11.6)	–0.02
L Fast cycles (Group 5 only)	4.0 (2.2)	−0.11*
L Slow cycles (Group 5 only)	5.1 (4.1)	0.08
L Hybrid technique cycles (Group 6 only)	5.1 (2.9)	0.01
L Technique min (all groups)	8.7 (9.2)	–0.02
L Min back to sleep (all groups)	19.5 (52.0)	–0.05

L, logbook variable. Group 1 = MILD + WBTB (no RT), Group 2 = MILD + WBTB + RT Breath, Group 3 = MILD + WBTB + RT Hands, Group 4 = MILD + WBTB (no RT), Group 5 = SSILD + WBTB (no RT), Group 6 = SSILD/MILD Hybrid + WBTB. All correlations are point-biserial and based on daily observations. *p = 0.05.

Participants turned on the light when they awoke to practice lucid dreaming techniques on 467 occasions (28.7%) as opposed to keeping the light turned off. A 2 × 2 Chi^2^ test showed that this was not related to lucid dreaming: χ^2^(1, *N* = 1626) = 0.30, *p* = 0.582, *V* = 0.01. Participants got out of bed after the alarm went off and before practicing lucid dreaming techniques on 1140 occasions (70.1%) as opposed to staying in bed. A 2 × 2 Chi^2^ test showed that this was not related to lucid dreaming: χ^2^ (1, *N* = 1624) = 1.08, *p* = 0.298, *V* = 0.03. Participants fell asleep while performing lucid dreaming techniques on 1162 occasions (70.7%). A 2 × 2 Chi^2^ test showed that this was not related to lucid dreaming: χ^2^(1, *N* = 1642) = 0.01, *p* = 0.966, *V* = 0.01.

A 2 × 2 Chi^2^ test was conducted to assess the hypothesis that lucid dreaming rates would be significantly higher when participants took 5 min or less to fall asleep after practicing lucid dreaming techniques compared to when they took more than 5 min to fall asleep. Mean Week 2 *L DRF Lucid* was 17.5% (*SD* = 38.1%) for 177 occasions when participants fell asleep within 5 min or less, compared to 13.8% (*SD* = 34.6%) for 275 occasions when participants took more than 5 min to return to sleep. However, this difference was not significant: χ*^2^*(1, *n* = 452) = 1.14, *p* = 0.286, *V* = 0.05. Therefore, these findings did not support the hypothesis. To further explore the hypothesis, another 2 × 2 Chi^2^ test was conducted using the criterion of 10 min or less instead of 5 min or less. Mean *L DRF Lucid* was 18.3% (*SD* = 38.7%) for 263 occasions when participants fell asleep within 10 min or less, compared to 11.1% (*SD* = 31.5%) for 189 occasions when participants took more than 10 min to return to sleep. This difference was statistically significant: χ^2^(1, *n* = 452) = 4.33, *p* = 0.037, *V* = 0.10. When this test was repeated for each of the six groups individually the results were non-significant in all cases. This may be due to insufficient statistical power.

### Additional Exploratory Analyses

Mann-Whitney tests were conducted to further explore factors related to the success rate of the lucid dream induction techniques and are presented in Table 6. On nights when participants were successful in inducing lucid dreams, they had significantly better sleep quality and significantly higher general dream recall compared to nights when they failed to induce lucid dreams. Participants in Group 5: SSILD + WBTB (no RT) also did more fast cycles on nights when they had lucid dreams. As noted in section “Relationships With Lucid Dreaming,” there was no significant correlation between *P Lucid tech freq* and Week 2 *L DRF Lucid*. Further to this, a Mann-Whitney test showed that there was no difference in Week 2 *L DRF Lucid* between participants who had prior lucid dream induction experience (*M* = 15.3%, *SD* = 24.9%) and participants without prior experience (*M* = 16.4%, *SD* = 25.7%): *Z*(355) = 0.75, *p* = 0.454, *r* = 0.04.

**TABLE 6:** Mann–Whitney tests for differences in week 2 logbook variables between nights when practice of lucid dream induction techniques was and was not followed by lucid dreaming.

**Week 2 Logbook variable**	**Lucid dreaming reported**			**No lucid dreaming reported**			**Mann–Whitney**		
	***n***	***M***	***SD***	***n***	***M***	***SD***	***Z***	***p***	***r***
L Reality tests (Groups 2 and 3)	44	9.8	4.0	350	9.7	4.0	0.48	0.629	0.02
L MILD phrase repetitions (Groups 1, 2, 3, and 4)	177	13.8	13.1	1130	13.5	11.4	0.65	0.514	0.02
L Fast cycles (Group 5 only)	58	4.4	4.1	276	4.0	1.5	2.07	0.039	0.11
L Slow cycles (Group 5 only)	58	6.8	8.0	276	4.7	2.6	1.46	0.145	0.08
L Hybrid technique cycles (Group 6 only)	41	5.5	3.9	293	5.0	2.8	0.19	0.852	0.01
L Technique min (all groups)	235	9.4	11.1	1406	8.6	8.9	0.85	0.398	0.02
L Min back to sleep (all groups)	69	17.8	29.4	383	19.8	55.1	1.05	0.293	0.05
L DC per day (all groups)	236	2.8	1.8	1406	1.7	1.5	9.33	<0.001	0.23
L DQ (all groups)	236	10.2	9.9	1406	5.2	6.5	10.54	<0.001	0.26
L Time asleep (all groups)	236	7.8	1.3	1402	7.7	1.3	0.56	0.576	0.01
L Sleep quality (all groups)	236	3.6	0.9	1405	3.4	0.9	2.08	0.037	0.05
L Tiredness on waking (all groups)	236	2.1	1.0	1405	2.3	1.1	1.81	0.070	0.05
L Sleep dep yesterday (all groups)	236	1.9	1.0	1405	2.0	1.1	1.44	0.150	0.04

*L, logbook variable. Group 1 = MILD + WBTB (no RT), Group 2 = MILD + WBTB + RT Breath, Group 3 = MILD + WBTB + RT Hands, Group 4 = MILD + WBTB (no RT), Group 5 = SSILD + WBTB (no RT), Group 6 = SSILD/MILD Hybrid + WBTB.*

## General Discussion

Participants of the International Lucid Dream Induction Study (ILDIS; *N* = 355) completed a pre-test questionnaire, a baseline Week 1 logbook period, and then practiced one of six different combinations of lucid dream induction techniques in Week 2. All six technique combinations were effective.

### Lucid Dream Induction Techniques

#### Reality Testing (RT)

No significant correlations were observed between number of RT performed each day and lucid dreaming incidence. This replicates the lack of significant correlations in the RT only and the RT + WBTB + MILD groups of the NALDIS, and the lack of correlation reported by Konkoly and Burke (2019). There was no significant difference in lucid dreaming rate between the MILD + WBTB groups that did and did not perform RT during the day. These findings are consistent with the NALDIS and studies by LaBerge (1988) and Taitz (2011), in which RT was ineffective. It remains possible that RT is effective over longer periods of time, as found for 3 weeks in studies by Purcell et al. (1986) and Purcell (1988), and 8 weeks in a study by Schlag-Gies (1992). Many participants complained that performing RT was burdensome and difficult to remember. This burden may reduce motivation and compliance with more effective techniques when practiced in combination. Lucid dream induction studies should avoid daytime RT unless this technique is of specific interest. The present author believes that RT is still a valuable technique for confirming whether one is dreaming, and as a specialized lucid dreaming practice for cultivating mindfulness, which is associated with lucid dreaming (Stumbrys et al., 2015).

#### The Mnemonic Induction of Lucid Dreams (MILD) Technique

The MILD technique was effective in four separate experimental groups, two of which involved performing RT during the day. As discussed above, the addition of RT did not result in higher lucid dreaming rates. The weighted average lucid dreaming rate for the four MILD technique groups was 16.5%. This is close to the success rate reported in the NALDIS of 17.4%. These findings replicate the NALDIS and several other studies that have shown the MILD technique to be effective (LaBerge, 1988; Levitan, 1989, 1990a,1990b,^1991^; Edelstein and LaBerge, 1992; Levitan et al., 1992; LaBerge et al., 1994, 2018; Levitan and LaBerge, 1994; Saunders et al., 2017; Konkoly and Burke, 2019). Although there were no statistically significant differences between the effectiveness of the hybrid SSILD/MILD technique and the other techniques in Cohort 2, results show that the overall lucid dreaming rate in Week 2, the improvement in week 2 compared to Week 1, and the effect size were all lowest for the SSILD/MILD hybrid group.

#### The Senses Initiated Lucid Dream (SSILD) Technique

The SSILD technique was shown to be effective, with a large effect size and a Week 2 lucid dreaming rate of 16.9%. This rate is almost identical to the weighted average rate for the four groups that practiced the MILD technique (*M* = 16.5%), as well as the RT + WBTB + MILD group of the NALDIS (*M* = 17.4%). These findings indicate that the SSILD technique is similarly effective for inducing lucid dreams as the MILD technique. There are several possible explanations for how the SSILD technique may induce lucid dreams. One is that repeatedly focusing attention on the visual, auditory and kinesthetic sensory modalities causes a generally increased awareness of perceptual stimuli that persists into REM sleep, making it more likely that the practitioner will notice that they are dreaming, either through generally increased awareness, or through recognition of anomalies within the dream. This could also occur if repeated sensory modality shifts persist upon entering REM sleep. Indeed, one participant reported: “as I was drifting off to sleep, I found myself continuing to do the technique, even though I wasn’t trying to.” Another possible explanation is that repeatedly refocusing one’s attention on different types of perceptual stimuli causes a general increase in cortical activation that increases the likelihood of lucid dreaming.

### Predictors and Effects of Lucid Dream Induction

#### Prior Technique Experience

There was no relationship between Week 2 lucid dreaming and whether participants had ever practiced a lucid dream induction technique, nor with the frequency of practice for those who did have prior experience. This indicates that MILD and SSILD combined with WBTB can be used successfully regardless of baseline lucid dreaming or prior technique experience.

#### General Dream Recall

In Week 2, lucid dreaming rates were significantly correlated with general dream recall rates. Pre-test lucid dreaming was also correlated with pre-test general dream recall. Furthermore, participants recalled significantly more dreams on nights when lucid dreaming occurred following technique practice. General dream recall was significantly lower in Week 2 compared to Week 1, indicating that the increased lucid dreaming rates cannot be attributed to simply recalling more dreams of all types. Taken together, these findings provide further support for the theory that superior general dream recall is conducive to lucid dreaming (see Aspy et al., 2017) and that general dream recall is a strong predictor of lucid dreaming (see Erlacher et al., 2014).

#### Technique Practice Variables

Lucid dreaming was not related to any of the variables that operationalized the way in which the lucid dream induction techniques were practiced, except for a weak correlation with the number of fast cycles in the SSILD + WBTB (no RT) group. The explanation for this correlation is unclear. Type 1 error is a likely possibility (*p* = 0.039).

#### Time Taken to Return to Sleep

In the NALDIS, lucid dreaming occurred 86.2% more often when participants fell asleep within 5 min of completing the MILD technique. This finding was not replicated in the ILDIS. However, upon further exploration, it was found that lucid dreaming occurred 64.9% more often on nights when participants of the ILDIS fell asleep within 10 min (*L DRF Lucid M* = 18.3%) compared to nights when they took more than 10 min (*L DRF Lucid M* = 11.1%). This effect is weaker than in the NALDIS. A possible explanation is that participants of the ILDIS were able to fall asleep more quickly in general due to being given suggestions for how to do this. Notwithstanding, findings from the ILDIS provide further support that lucid dreaming techniques are more effective when one can return to sleep quickly. For the MILD technique, this probably makes it more likely that the mnemonic intention to remember that one is dreaming will be recalled during REM sleep. For the SSILD technique, it may be due to increased cortical activation and/or increased awareness of perceptual stimuli being more likely to persist into REM sleep.

#### Effects of Lucid Dream Induction on Sleep

Sleep quality was superior on nights when participants successfully induced lucid dreams compared to nights when they failed to induce lucid dreams. Participants also reported significantly more time asleep and significantly less tiredness on waking in Week 2 compared to Week 1. These findings indicate that sleep quality was not adversely affected by successful induction of lucid dreams but may have been adversely affected by unsuccessful attempts. This would be expected if the probability of success is related to the amount of time taken to return to sleep. These findings are consistent with findings from the NALDIS, whereby successful lucid dream induction using the MILD technique was related to the amount of time taken to return to sleep and did not adversely affect sleep quality. Vallat and Ruby (2019) have recently drawn attention to the fact that increasing the frequency of lucid dreams may have unknown negative impacts on the usual processes that occur during REM sleep, due to the fact that lucid dreaming involves a brain state that is neurologically distinct from non-lucid REM sleep. They also raised concerns about potential negative health impacts of the sleep disruption inherent in many lucid dreaming techniques. Soffer-Dudek (2020) raised similar concerns about the effects of lucid dreaming on sleep as well as potential disruptions to reality-fantasy boundaries, which may be of particular concern to clinical populations with disorders such as pscyhosis. More research is needed to investigate the impacts of lucid dreaming generally, and lucid dreaming training specifically, on sleep quality.

### Strengths and Limitations

Strengths include the wide range of measures used, the use of measures that operationalized the way in which lucid dream induction techniques were practiced, the comparison of six different lucid dream induction technique combinations, and the large and highly diverse international sample of participants that were mostly employed non-students (71.8%), with nearly equal proportions of people who did (54.9%) and did not (45.1%) have prior lucid dreaming technique experience. Indeed, the ILDIS is the largest study of lucid dream induction techniques to date. As with the NALDIS, the ILDIS has high ecological validity. Participants practiced the techniques in their own homes using written instructions, which reflects how cognitive lucid dream induction techniques are usually practiced. A limitation of the ILDIS is the high attrition rate from the initial sample that completed the pre-test questionnaire (*N* = 1618) to the final sample (*N* = 355). Findings are likely to be most generalizable to people who are highly motivated to learn lucid dreaming. The use of self-report measures is a potential limitation to the findings that lucid dream induction did not adversely affect sleep quality. This is because the excitement of having a lucid dream may have counteracted feelings of tiredness upon waking. Another limitation is that the large number of statistical tests increases the familywise error rate. Results that are only marginally significant should therefore be interpreted with caution.

### Directions for Future Research

Further research is needed to gain a deeper understanding of the mechanisms through which the MILD and SSILD techniques work. This may yield potential avenues for refinement. One approach could be to ask participants to describe in detail exactly how they become lucid in each lucid dream, including whether they thought about or practiced the techniques in their dreams prior to becoming lucid. Sleep laboratory research could investigate whether the SSILD technique causes increased cortical activation and whether this activation is correlated with lucid dreaming. Further research is also needed to investigate the effectiveness of practicing the MILD, SSILD and RT techniques over longer periods of time than the single week used in the present study, and the effects of lucid dreaming training on sleep quality.

Findings provide further evidence that superior general dream recall is conducive to lucid dreaming. Thus, it may be possible to increase the effectiveness of cognitive lucid dream induction techniques using drugs and supplements that enhance dream recall. In a small pilot study by Ebben et al. (2002), ingestion of vitamin B6 (pyridoxine hydrochloride) prior to sleep was found to significantly enhance dream recall compared to placebo. In a larger replication study (Aspy et al., 2018), participants recalled 64.1% more dream content when they took 240 mg of vitamin B6 directly before bed compared to placebo. Future research should compare the effectiveness of cognitive lucid dream induction techniques both with and without vitamin B6 before bed.

Currently, the most evidence-based substance for inducing lucid dreams is Galantamine, a widely used and well-tolerated acetylcholine-esterase inhibitor that influences the REM-on neurotransmitter acetylcholine (LaBerge, 2004; Yuschak, 2006; Sparrow et al., 2016, 2018; LaBerge et al., 2018). In the most recent study by LaBerge et al. (2018), lucid dreaming occurred on 42% of nights when participants ingested 8 mg of Galantamine in addition to practicing the MILD technique and, in most cases, using an external LED light stimulation device. According to Yuschak (2006), Galantamine is more effective when combined with Alpha-GPC, a form of choline that acts as a precursor to acetylcholine. It may be even more effective to take vitamin B6 before bed and then a combination of Galantamine and Alpha-GPC during a WBTB period 5 h after going to sleep, before practicing a cognitive lucid dream induction technique such as MILD or SSILD and then returning to sleep within 5–10 min. An external light stimulation device may further increase the success rate (see Mota-Rolim et al., 2019). This combination of cognitive, pharmacological and external stimulation techniques is currently the most promising approach to lucid dream induction.

Future studies should operationalize the way in which lucid dream induction techniques are practiced, use valid and reliable measures of dream recall, and avoid the many methodological limitations of prior lucid dream induction studies (see Stumbrys et al., 2012; Aspy et al., 2017). These methodological issues – especially the inconsistency in the way that lucid dreaming rates are operationalized – are a major impediment to research progress. The present author implores other researchers to, at minimum, report the *L DRF Lucid* rate based on daily logbook observations in all lucid dream induction studies, so that the effectiveness of techniques can be determined and compared (see section “Materials”).

## Conclusion

Findings provide the strongest evidence to date that the MILD technique is effective for inducing lucid dreams. Findings indicate that the SSILD technique is similarly effective. In contrast, RT appears to be an ineffective lucid dream induction technique – at least for short periods such as 1 week in the present study.

## Data Availability Statement

The datasets generated for this study are available on request to the corresponding author.

## Ethics Statement

The studies involving human participants were reviewed and approved by the School of Psychology Human Research Ethics Committee at the University of Adelaide. The patients/participants provided their written informed consent to participate in this study.

## Author Contributions

DA-H was the sole author of this study and was solely responsible for all tasks involved. This includes experiment design, experiment management, data collection, data analysis, literature review, and manuscript authorship.

## Conflict of Interest

The author declares that the research was conducted in the absence of any commercial or financial relationships that could be construed as a potential conflict of interest.
